# Nature of Dielectric Response of Phenyl Alcohols

**DOI:** 10.1021/acs.jpcb.3c02335

**Published:** 2023-07-03

**Authors:** Magdalena Tarnacka, Anna Czaderna-Lekka, Zaneta Wojnarowska, Kamil Kamiński, Marian Paluch

**Affiliations:** † August Chełkowski Institute of Physics, University of Silesia in Katowice, 75 Pułku Piechoty 1, 41-500 Chorzów, Poland

## Abstract

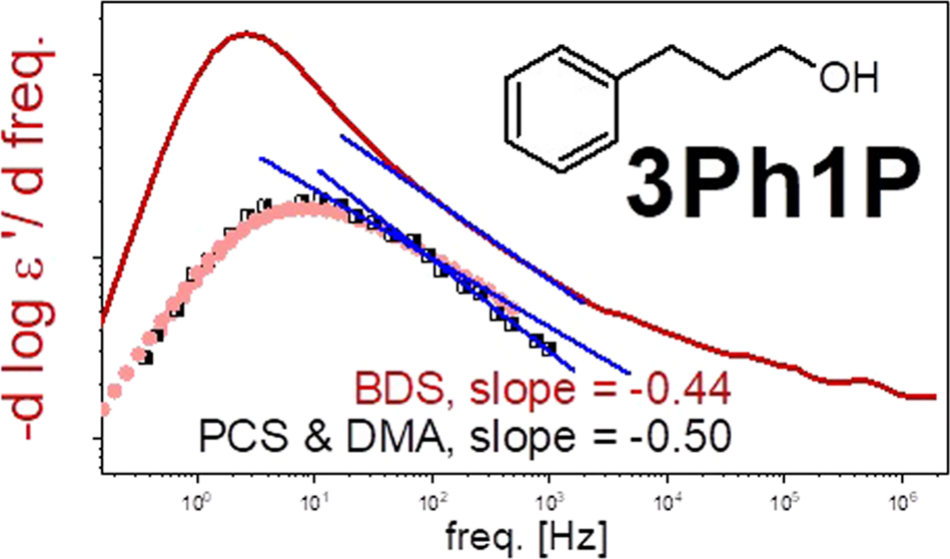

Phenyl alcohols (PhAs) are an interesting class of materials, for which the dielectric response reveals only the presence of single prominent Debye-like (*D*) relaxation, interpreted as a *genuine* structural (α) process. Herein, we have performed dielectric and mechanical measurements on a series of PhAs characterized by the varying length of the alkyl chain and found that this interpretation is not valid. Analysis of the derivative of the real part of the complex permittivity together with the mechanical and light scattering data clearly indicated that the prominent dielectric *D*-like peak is actually a superposition of both cross-correlation between dipole–dipole (*D*-mode) and self-dipole correlation (α-process) and that the distinguished α-mode exhibits a similar (“*generic*”) shape of PhAs independently to their molecular weight and applied experimental technique. Therefore, the data presented herein contribute to the whole discussion focused on the dielectric response function and universality (or diversity) of the *spectral* shape of the α-mode of polar liquids.

## I Introduction

The common feature of the majority of supercooled liquids is both their nonlinear temperature dependence of the structural (α) dynamics and the nonexponential shape of the α-peak. Moreover, for some particular cases, the Debye (*D*) mode of a slower dynamics than the α-process can be additionally seen in their dielectric response due to the formation of supramolecular structure as a result of, i.e., van der Waals or Columbic interactions or hydrogen bonding.^1−12^ This characteristic response is usually observed for monohydroxyl alcohols (MA, able to form excessive hydrogen bonding networks),^13−16^ where it often almost completely dominated dielectric spectra (the α-mode can be detected as an excess wing in the high-frequency region). Surprisingly, a totally different scenario was observed in the case of phenyl-substituted MA (phenyl alcohols, PhAs).^17^ For this specific class of materials, dielectric loss spectra exhibit only the presence of a Debye-like process (characterized by the Kohlrausch–Williams–Watts (KWW) stretched exponent, *β*_*KWW*_ ≈ 0.90).^18−21^ As the calorimetric glass transition temperature, *T*_*g*_, agrees with the one determined from dielectric relaxation times, τ, of this mode, this single dominant dielectric relaxation observed in the case of PhAs was interpreted as a *genuine α*-mode (manifesting as a narrow peak due to their high static permittivity).^20^ However, recent studies on phenyl-substituted propanol by means of dielectric and photon correlation spectroscopy (PCS) have clearly shown that the dielectric Debye-like mode observed in loss spectra is, in fact, the superposition of two processes (the structural and prominent Debye) visible as only one relaxation peak due to their similar time scales.^22,23^ The combination of dielectric and light scattering data enabled authors to disentangle the contributions of the two types of dynamics, governed by self-dipole correlation and cross-correlation between dipole–dipole.^14,24^ Moreover, it was postulated that the dielectric response function is dominated by the latter contributions (Debye process), while the former one is of lower importance (α-mode). Considering the high Kirkwood factor, *g*_*K*_, of phenyl alcohols (much larger than unity^19,25^), one can recall recent works showing that as the *g*_*K*_ parameter or polarity increases, the cross-correlation between dipoles dominate the dielectric response function.^26−28^

**Scheme 1:**
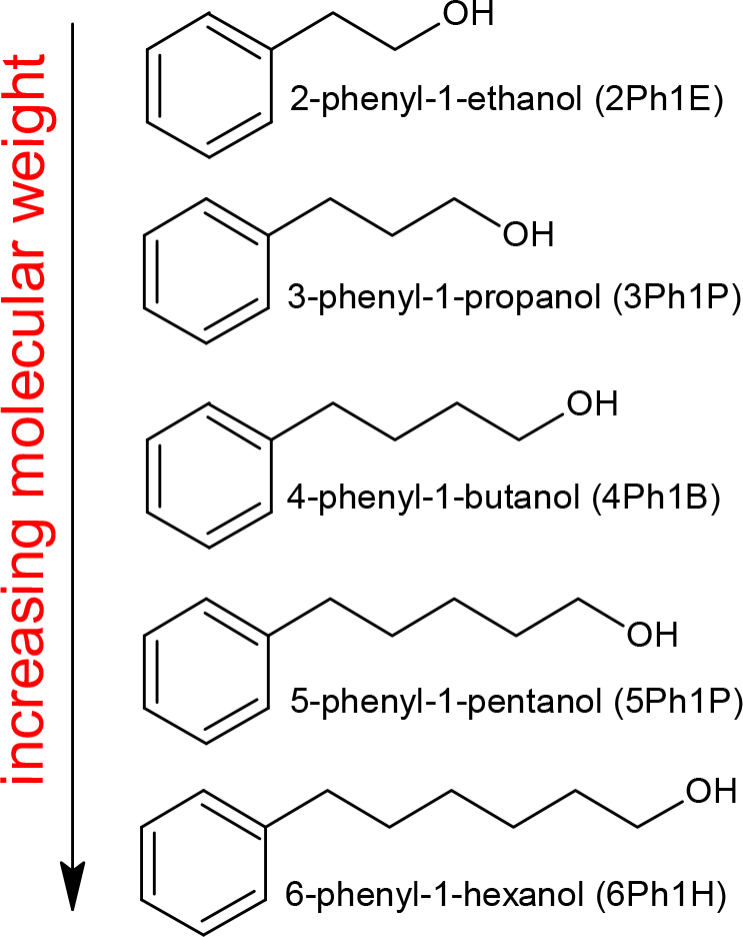
Chemical Structures of Investigated Phenyl Alcohols

In this study, we report, for the first time, mechanical data collected for a series of phenyl-substituted primary monohydroxy alcohols (PhAs) with varying alkyl chain lengths (from ethanol to hexanol; Scheme 1) together with the analysis of the derivative of the real part of permittivity as a new contribution to the discussion concerning the dielectric response of PhAs. Furthermore, the data collected were used in the context of a more general phenomenon, which is *the universality (or diversity) of the relaxation shape of the alpha mode of polar liquids*. This issue is an ongoing hot discussion on the *generic*(14) or non-*generic*^29,30^ shape of the structural process seen by different experimental methods.

## II Materials and Methods

### Materials

A set of phenyl-substituted primary monohydroxy alcohols (PhAs) of purity higher than 97% were purchased from Sigma-Aldrich, 2-phenyl-1-ethanol (2Ph1E), 3-phenyl-1-propanol (3Ph1P), 4-phenyl-1-butanol (4Ph1B), and 5-phenyl-1-pentanol (5Ph1P), and Acros Organics, 6-phenyl-1-hexanol (6Ph1H). The structure of the investigated material is shown in Scheme 1. Prior to dielectric and mechanical investigations, the materials were heated at 50 °C for at least 30 min using vacuum to ensure there was no water in the system. Freshly purified samples were immediately measured as a function of temperature.

### Differential Scanning Calorimetry (DSC)

Calorimetric measurements were carried out by a Mettler-Toledo DSC apparatus equipped with a liquid nitrogen cooling accessory and an HSS8 ceramic sensor (heat flux sensor with 120 thermocouples). Temperature and enthalpy calibrations were performed using indium and zinc standards. The sample was prepared in an open aluminum crucible (40 μL) outside of the DSC apparatus. Samples were scanned at various temperatures at a constant heating rate of 10 K/min. Representative calorimetric curves obtained for 2Ph1E, 4Ph1B, and 6Ph1H are shown in Figure S1.

### Broadband Dielectric Spectroscopy (BDS)

Dielectric measurements in the frequency range from 10^–1^ to 10^6^ Hz were carried out using the Novocontrol spectrometer, equipped with an Alpha Impedance Analyzer. The temperature was controlled using a nitrogen gas cryostat, Quatro Cryosystem, with a stability better than 0.1 K. All of the samples were sandwiched between two stainless-steel electrodes (diameter: 15 mm), distanced with two glass spacers (thickness: 100 μm), sealed within a Teflon ring, and placed inside the temperature-controlled sample cell. BDS measurements were carried out in a wide range of temperatures (*T* = 170–298 K). Note that samples were cooled from the liquid state below its calorimetric glass transition temperature, *T*_*g*_. However, in the case of 6Ph1H, the sample was measured in two different temperature regimes upon both (1) heating of quenched material and (2) cooling from room temperature due to an ongoing crystallization.

### Mechanical Investigations

The rheological measurements were conducted by using an Ares-G2 rheometer (TA Instruments). Oscillatory shear deformation was applied with controlled deformation amplitude, which was kept in the range of the linear viscoelastic response. Parallel plate geometry was used with a plate diameter of 4 mm. Values of storage (*G′*) and loss (*G″*) shear modulus as a function of radial frequency ω in the range from 0.1 to 100 rad s^–1^ (12 points per decade) and over the temperature range from *T* = 177 K to *T* = 293 K were obtained at various constant temperatures maintained to within ± 0.1 K. In the temperature range, where the *G*″ peaks are not visible, the time–temperature superposition (TTS) rule can be applied to determine segmental relaxation times, *τ*_*α*_. According to this criterion, *G*′ and *G*″ spectra collected under various temperature conditions form the master curve when shifted horizontally by the shift factor, *α*_*T*_, to superimpose at the chosen reference temperature. Therefore, *τ*_*α*_ is determined according to the following equation: log(*τ*_*α*_(*T*)) = log(τ(*T*_*ref*_)) + log(*α*_*T*_). Note that τ(*T*_*ref*_) indicates the segmental relaxation time calculated by the following equation, τ = 1/(2*πf*), where *f* is a frequency of the *G*″(ω) maximum at a chosen *T*_*ref*_. In the temperature range where the *G*″ peaks are not visible, the Maxwell equation, taking into account the infinite-frequency glassy shear modulus, *G*_∞_, of the liquid, was applied to determine *τ*_*α*_.

## III Results and Discussion

**Figure 1:**
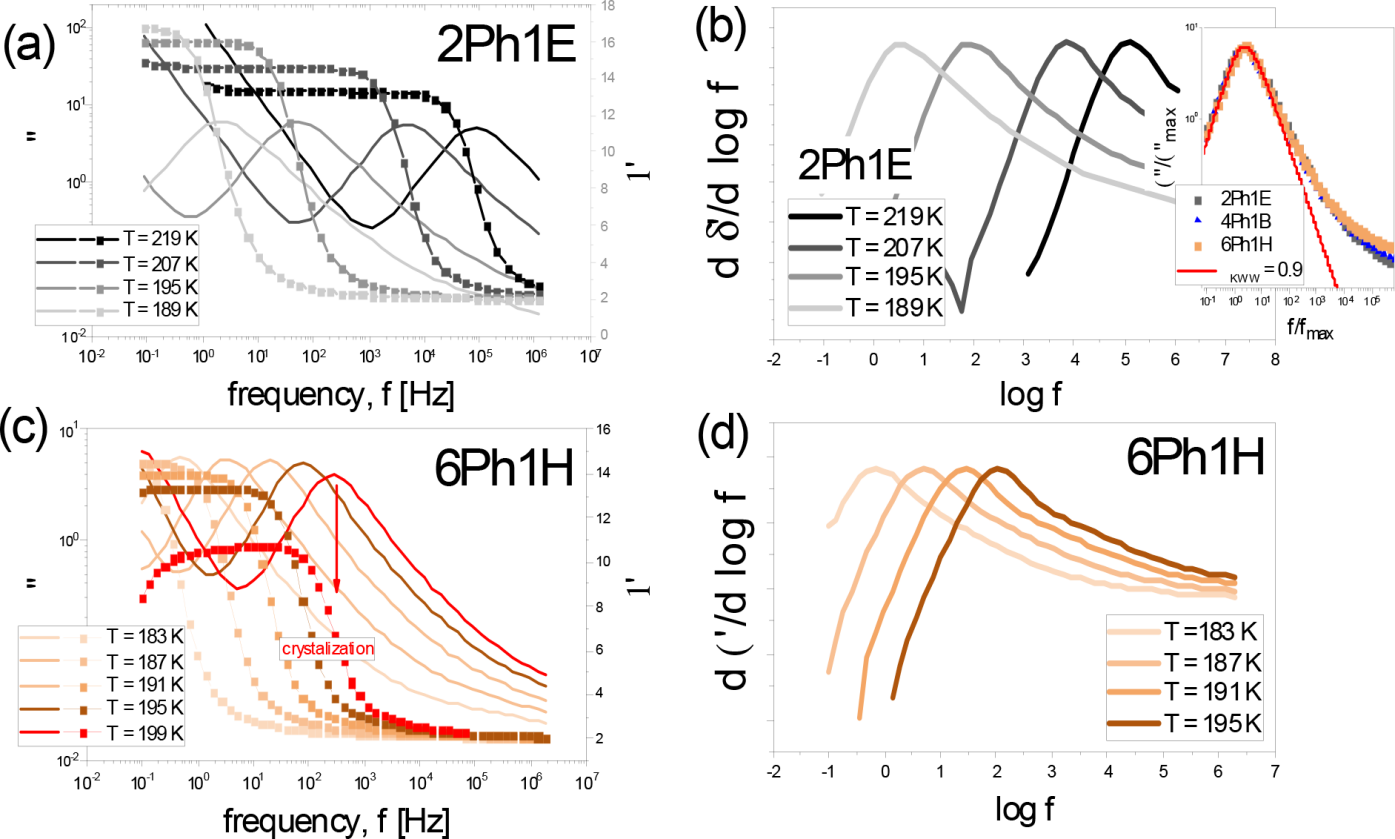
(a, c) Real, *ε*′, and imaginary, *ε*″, part of permittivity measured for 2Ph1E and 6Ph1H above *T*_*g*_. A decreasing amplitude of both *ε*′ and *ε*″ observed in 6Ph1H with increasing temperature indicates ongoing crystallization, which explains a limited set of dielectric data recorded for this compound. (b, d) derivatives of *ε*′ of 2Ph1E and 6Ph1H. As the inset in panel b, the comparison of dielectric loss spectra of chosen PhAs at constant *τ*_*D*_ near *T*_*g*_ is shown.

Representative real, *ε*′, and imaginary, *ε*″, parts of permittivity measured for 2-phenyl-1-ethanol (2Ph1E) and 6-phenyl-1-hexanol (6Ph1H) above their glass transition temperatures, *T*_*g*_, are presented in Figure 1a,c. As can be seen, the dielectric loss spectra of examined PhAs revealed two processes: the dc conductivity and the single dominant relaxation process; both shifting to lower frequencies with lowering temperature. Note that the same situation is also noted in other examined PhAs. One can add that fitting the prominent loss peak of all studied herein alcohols to the KWW function yields *β*_*KWW*_ ∼ 0.90.^19,21^ This indicates that all examined PhAs exhibit the similar shape of the dominant process, independent of their molecular weight. This fact is well-illustrated in the inset in Figure 1b, where the loss peaks recorded for chosen materials and characterized by the same τ are compared. Taking into account that the value of the stretching parameter is close to unity (*β*_*KWW*_ ∼ 0.90), we will label the dominant relaxation process as a Debye-like mode in the further part of this paper. Nevertheless, it should be mentioned that the combination of dielectric and light scattering data collected for a series of phenyl-substituted propanols^14^ clearly indicated that the observed loss peak might be, in fact, composed of the two contributions originating from the self- and cross-correlations of the dipoles with the latter dynamics dominating over the former one for the materials having *g*_*K*_ ≫ 1 (as in the case of PhA). Having in mind these results and recent paper by Arrese-Igor et al.,^31^ we decided to show the measured data in the derivative representation, i.e.: −d(log *ε*′)/d(log *freq*.) vs log(*freq*.). Surprisingly, as shown in Figure 1b,d, this simple mathematical operation clearly demonstrated that there is an additional process being faster than the Debye-like process; see Figure S2a. Moreover, interestingly, the separation between both (α and *D*) modes observed in Figure 1b,d seems to increase with the elongation of the alkyl chain, as the structural relaxation is more resolved from the *D*-mode for 6Ph1H when compared to 2Ph1E (see Figure S2b,c). One can add that this set of data mimic those often reported for various MA, where the α-process is observed in loss spectra as an excess wing.^13−16^

As clearly shown above, the dielectric Debye-like mode in loss spectra might be, in fact, the superposition of two processes (the structural and prominent Debye) but observed as only one relaxation peak due to their similar time scale.^14,19^ Thus, to confirm this scenario and follow the structural dynamics, we performed complementary mechanical measurements.

**Figure 2:**
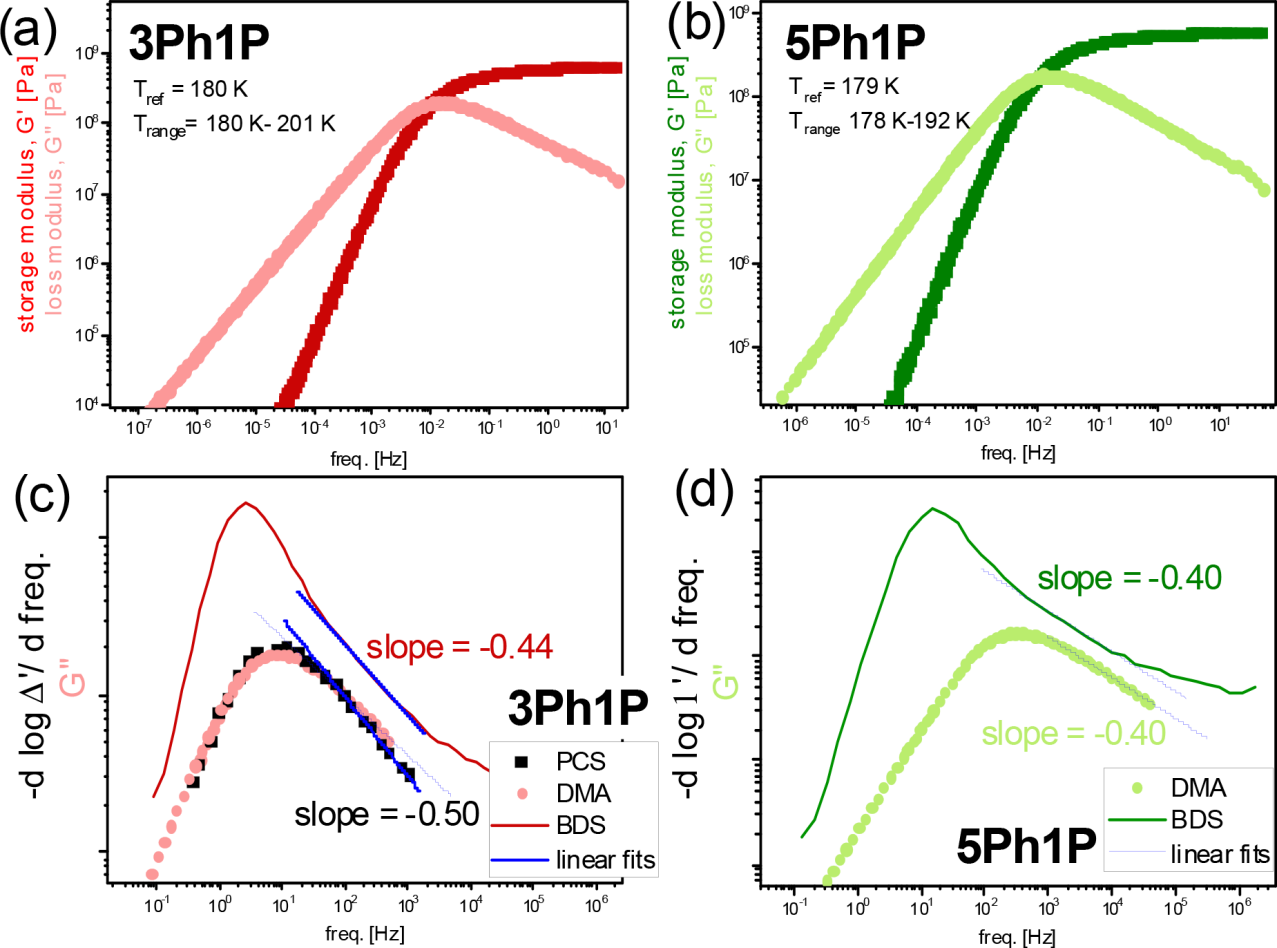
(a, b) Master curves of measured storage, *G*′, and loss, *G*″, modulus of 3Ph1P (a) and 5Ph1P (b) superimposed at chosen reference temperature, *T*_*ref*_. (c, d) Comparison of spectra recorded by various experimental techniques at the indicated temperature conditions. PCS data for 3Ph1P taken from ref (14).

Figure 2a,b demonstrates master curves of measured storage, *G*′, and loss, *G*″, modulus for 3Ph1P (a) and 5Ph1P (b). The presented figure was constructed from the frequency dependencies of *G*′ and *G*″ measured within the range of 0.1–100 rad s^–1^ at various temperatures (Figure S3), which were shifted horizontally by the shift factor, *α*_*T*_, to superimpose at chosen reference temperature, *T*_*ref*_. The *α*_*T*_(*T*)-dependences for all examined PhAs are shown in Figure S4a. As can be seen in Figure 2a,b, the mechanical loss spectra revealed a single-peak structure, indicating structural (α) relaxation (of local viscous flow processes). Interestingly, both storage, *G*′, and loss, *G*″, modulus are described by the power law *G*′(*f*) ∝ *f*^2^ and *G*″(*f*) ∝ *f*^1^ in the low frequency regime (see red solid lines in Figures 2a,b and S4c,d).^32^ At this point, we would like to stress two issue. First, the shape of the mechanical α-process is the same for all examined PhAs independently of their molecular weight (Figure S4d). This result agrees very well with the PCS data obtained earlier for a series of phenyl propanols by Böhmer et al.^14^ Second, normalized shear viscosity revealed viscoelastic behavior expected for nonassociating liquids; see Figure S4b. It is quite surprising result as many mechanical data available in the literature for various MA clearly report an emerging of additional relaxation process slower than the α-mode (or a crossover from an intermediate to the terminal power law) for these materials.^33−37^ This process was considered as evidence of an additional slow dynamics of supramolecular structures (analogous to the dielectric Debye relaxation). One can assume that the observed herein behavior typical for nonassociating liquids might be a result of the presence of phenyl group, leading to the formation of relatively small associates (which do not affect mechanical properties of examined PhAs).^21,38^ Alternatively, one can postulate that this additional mechanical process might be not resolved due to a time scale similar to that of the α-mode. Note that studies on a series of octanol structural isomers (*x*-methyl-3-heptanol, where *x* = 2–6) shown that the separation between both observed mechanical processes increases with an increasing distance between the methyl and hydroxyl groups (due to affecting the morphology, but not population, of associates).^37^

Furthermore, in Figure 2c,d, we compared the normalized dielectric and shear- mechanical loss spectra for 3Ph1P and 5Ph1P. On the other hand, in case of 3Ph1P, we added the light scattering data PCS data for 3Ph1P that were taken from ref (14). Note that the presented spectra were obtained at the same temperatures. Taking into account data shown in Figures 2c,d, we would like to highlight two issue. First, the position of the α-mode resolved from both light scattering and mechanical measurements agrees perfectly with the excess wing observed in the derivative of *ε*′ for all examined PhAs (Figure 2c). This observation clearly confirmed the appearance of the structural process in the dielectric data shown in Figure 1. Second, a good agreement between the shape of the α-process followed by various techniques can be well seen. Surprisingly for 3Ph1P, we observed that the slope of the excess wing (slope = −0.44) due to contribution of the structural process agrees almost perfectly with the one determined for the high-frequency flank of the peak observed in mechanical and also in the light scattering measurements (slope = −0.50; see Figure 2c). This observation might indicate a universal (“*generic*”) spectral shape of structural dynamics independently to the applied experimental technique.^14,23,24^

**Figure 3:**
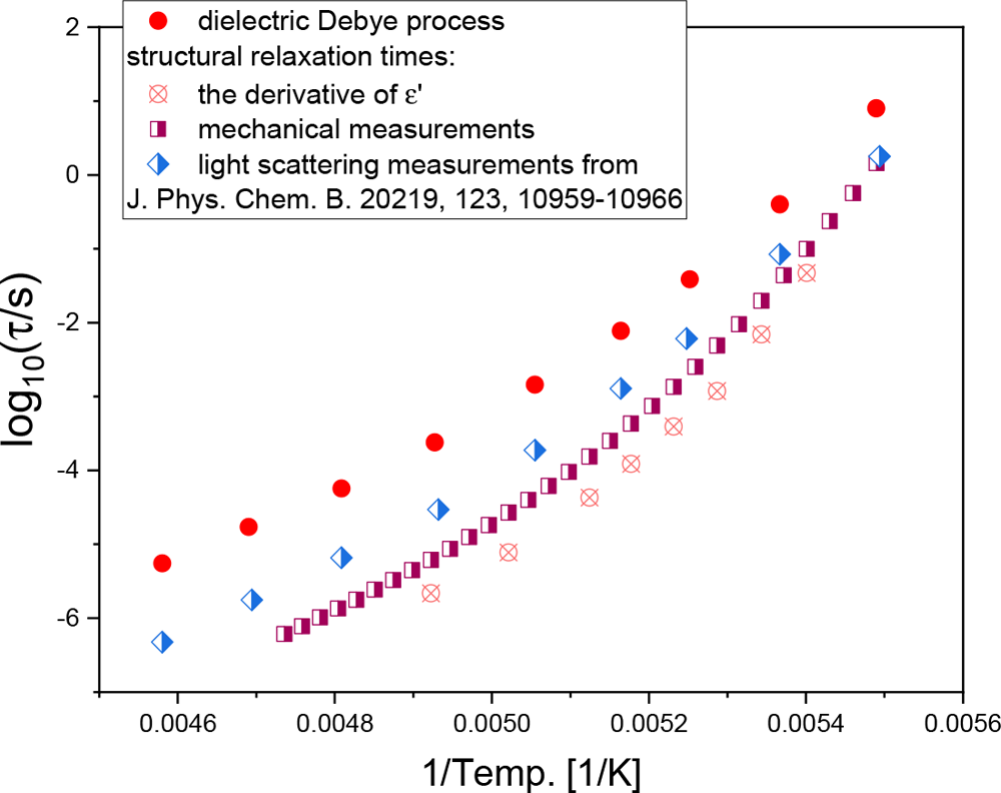
τ(*T*)-dependences were determined for 3Ph1P from different experimental techniques. PCS data for 3Ph1P were taken from ref (14).

Lastly in Figure 3, we compared the temperature dependences of structural, *τ*_*α*_, and Debye-like, *τ*_*D*_, relaxation times determined for 3Ph1P from various experimental techniques. Note that in case of dielectric data, we analyzed of the derivative representation of *ε*′ (as shown in Figure 1) with the superposition of two Havriliak–Negami (HN) functions.^39^ On the other hand, mechanical *τ*_*α*_ is determined according to the following equation: log(*τ*_*α*_(*T*)) = log(τ(*T*_*ref*_)) + log(*α*_*T*_), where τ(*T*_*ref*_) indicates the segmental relaxation time calculated by the following equation, τ = 1/(2*πf*), and *f* is a frequency of *G*″(ω) maximum at chosen *T*_*ref*_. The temperature dependences of shift factors determined for all examined PhAs are shown in Figure S4a. In the temperature range, where the *G*″ peaks are not visible, the Maxwell equation, taking into account the infinite-frequency glassy shear modulus, *G*_∞_, of the liquid, was applied to determine *τ*_*α*_. As shown in Figure 3, it was found that the relaxation times of the dielectrically active faster process observed in Figure 1 agree well with the *τ*_*α*_(*T*)-dependence determined from rheological measurements when approaching *T*_*g*_. Note that the dielectric *τ*_*α*_(*T*) values are significantly faster than those determined from PCS measurements; however, it should be mentioned that due to both (i) the dominant presence of the *D*-mode and (ii) the weak separation between both (*D* and α) processes, the accurate determination of dielectric *τ*_*α*_ is rather impossible. The observed convergence between *τ*_*α*_(*T*) shown in Figure 3 seems to confirm that all relaxation modes compared in Figure 2c,d are the structural relaxation and display a *generic* behavior.

## IV Conclusion

By comparing dielectric and mechanical response for a series of phenyl alcohols, it was possible to observe with no doubt that a single dominant relaxation observed in the dielectric response of PhAs is not a *genuine α*-mode,^20^ but rather this process is, in fact, the superposition of two processes (a slow Debye-like and α-one, resulting from both cross correlation between dipole–dipole and self-dipole correlation, respectively) but observed as only one relaxation peak due to their similar time scale.^14^ Moreover, it was clearly shown that the distinguished α-mode exhibits a similar (*generic*) shape for all studies PhAs independently of their molecular weight and applied experimental technique. Our finding is in agreement with data previously published for primary MA (including phenyl-substituted propanols) monitored by means of light scattering and dielectric spectroscopy, which showed that the shape of the α-relaxation remains the same among the investigated alcohols, irrespective of their chemical structure and possibly the architecture and the size of nanoassociates.^14^

## Supporting Information Available

Additional figures and tables, including DSC curves, the derivative presentation of *ε*′, frequency dependencies of *G*′ and *G*″, the temperature dependence of the shift factor, *α*_*T*_, obtained for all examined PhAs, the normalized complex viscosity, η*, recorded for all alcohols, and the comparison of master curves of measured *G*′ and *G*″ (PDF)
