# Supplementary material for: Nature of Dielectric Response of Phenyl Alcohols
Source: J Phys Chem B. 2023 Jul 3;127(27):6191–6. doi: 10.1021/acs.jpcb.3c02335 (PMC10350960; doi:10.1021/acs.jpcb.3c02335)
Supplement: Supplementary file 1 — jp3c02335_si_001.pdf [file jp3c02335_si_001.pdf]

Supporting information

On the Nature of Dielectric Response of Phenyl Alcohols.

Magdalena Tarnacka[#]*, Anna Czaderna-Lekka[#],

Zaneta Wojnarowska[#], Kamil Kamiński[#]*, Marian Paluch[#]

[#] *August Chełkowski Institute of Physics, University of Silesia in Katowice, 75 Pułku Piechoty 1, 41-500 Chorzów, Poland*

*Corresponding author: (MT) magdalena.tarnacka@us.edu.pl, (KK) kamil.kaminski@us.edu.pl

Table of content

Figure S1. Representative calorimetric curves recorded for 2Ph1E (black line), 4Ph1B (blue) and 6Ph1H (orange). 2

Figure S2. (a) The comparison of the original dielectric loss spectra with the results of the derivative analysis of the permittivity for 6Ph1H at T = 187 K; (b,c) The derivative presentation of *ε*' shown at the similar Debye relaxation times. 2

Figure S3. The frequency dependencies of *G*" measured for (a) 2Ph1E and (b) 5Ph1P. 3

Figure S4. (a) The temperature dependence of the shift factor, α_T, obtained for all examined PhAs; (b) the normalized complex viscosity, *η**, recorded for all alcohols; (c, d) the comparison of master curves of measured storage, *G*' (c), and loss, *G*" (d), modulus of all materials. 4

References: 4

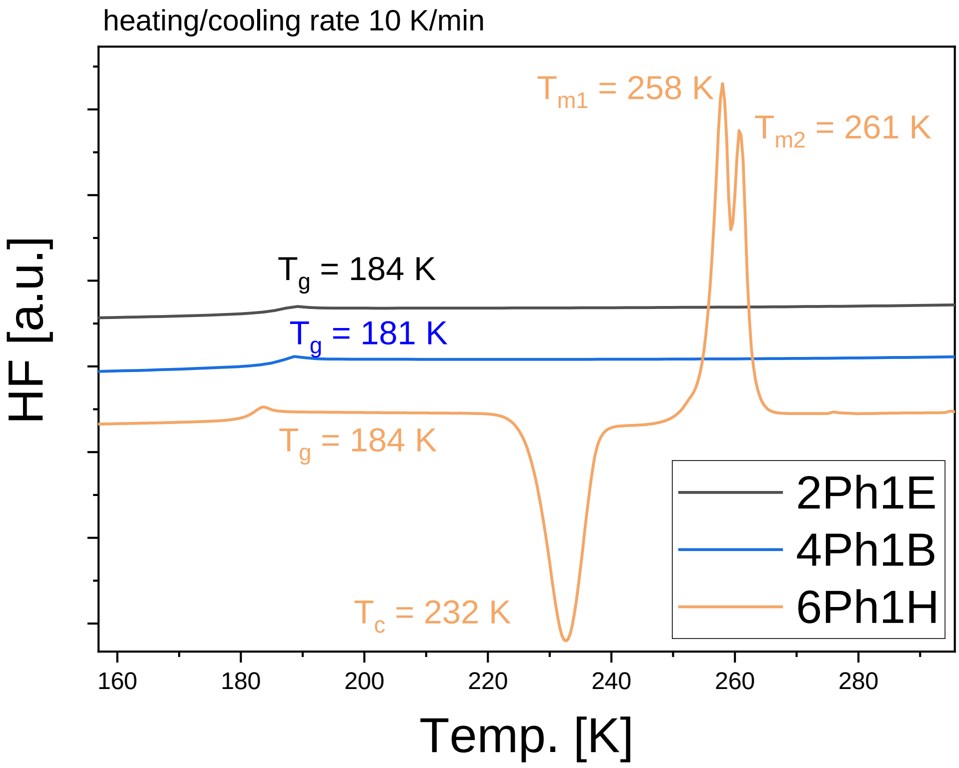

Figure S1. Representative calorimetric curves recorded for 2Ph1E (black line), 4Ph1B (blue) and 6Ph1H (orange).

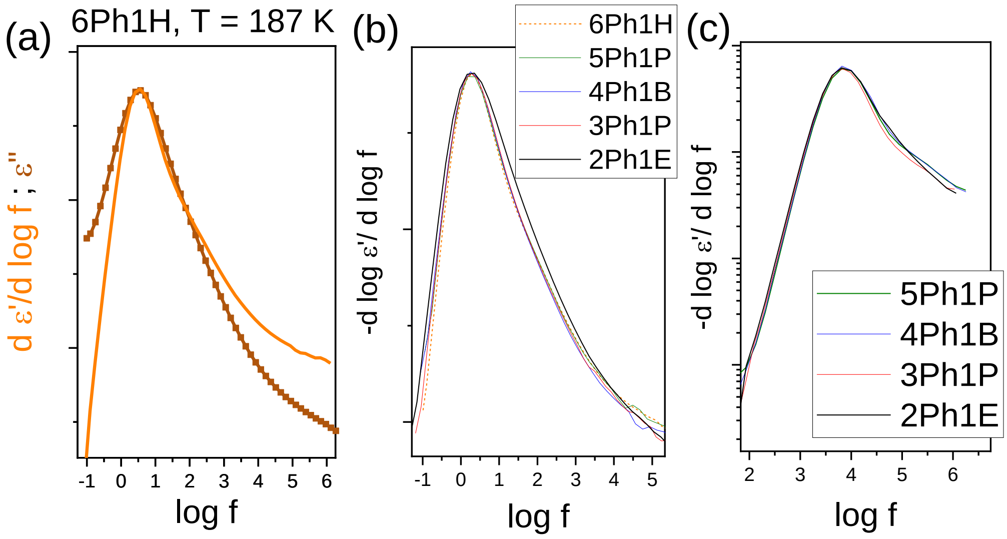

Figure S2. (a) The comparison of the original dielectric loss spectra with the results of the derivative analysis of the permittivity for 6Ph1H at T = 187 K; (b,c) The derivative presentation of ε' shown at the similar Debye relaxation times.

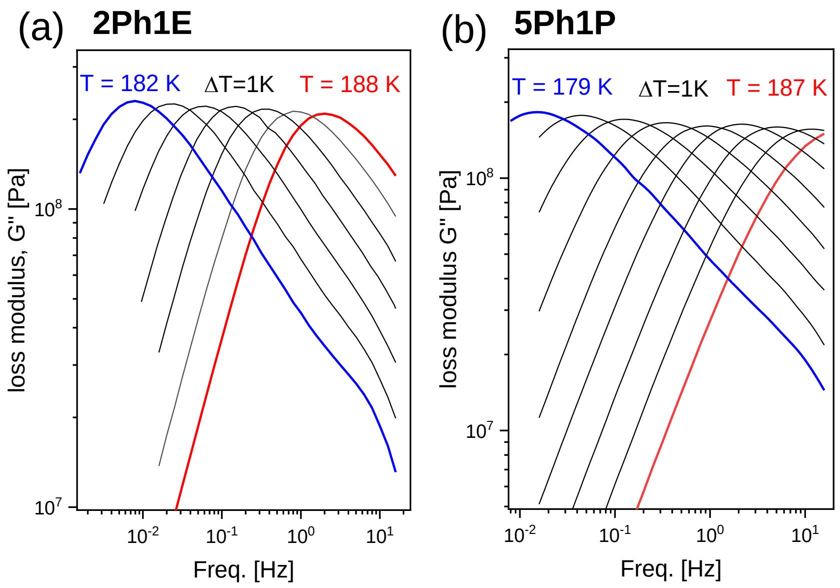

Figure S3. The frequency dependencies of *G*" measured for (a) 2Ph1E and (b) 5Ph1P.

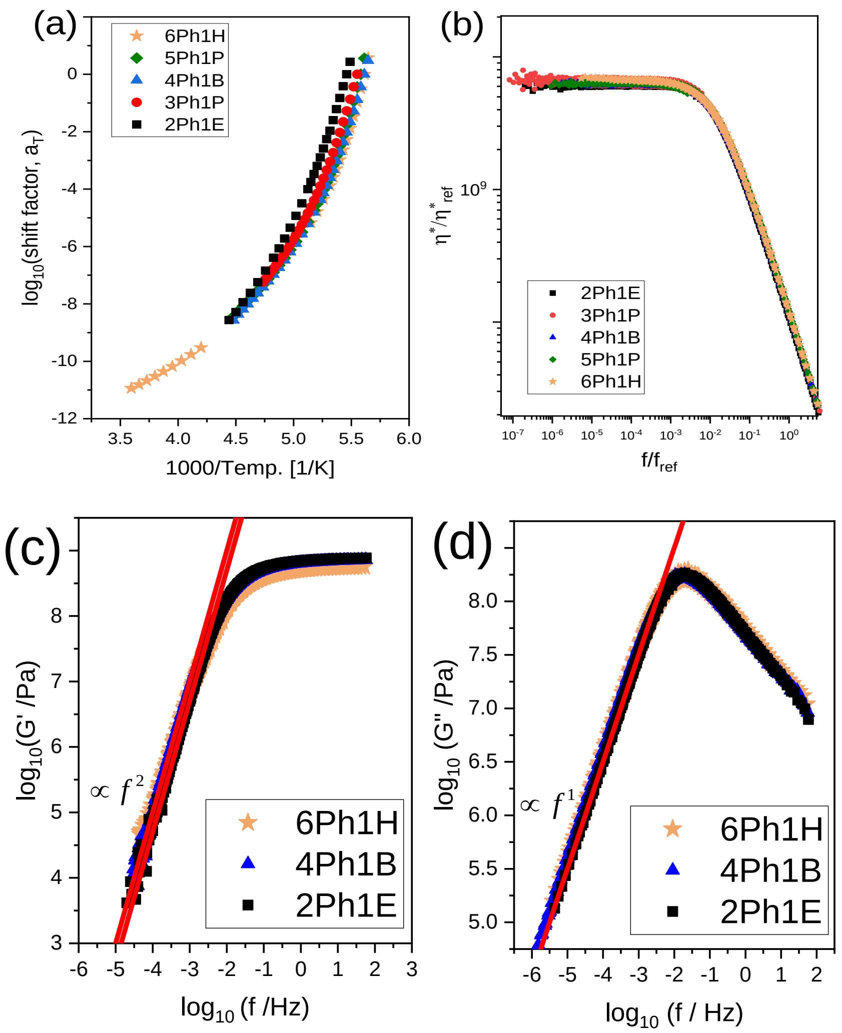

Figure S4. (a) The temperature dependence of the shift factor, α_T, obtained for all examined PhAs; (b) the normalized complex viscosity, η^*, recorded for all alcohols; (c, d) the comparison of master curves of measured storage, *G*' (c), and loss, *G*'' (d), modulus of all materials.

References:

(1) Soszka, N.; Hachuła, B.; Tarnacka, M.; Kamińska, E.; Grelska, J.; Jurkiewicz, K.; Geppert-Rybczyńska, M.; Wrzalik, R.; Grzybowska, K.; Pawlus, S.; Paluch, M.; Kamiński, K. The Impact of the Length of Alkyl Chain on the Behavior of Benzyl Alcohol Homologues – the Interplay between Dispersive and Hydrogen Bond Interactions. *Physical Chemistry Chemical Physics* **2021**, *23* (41), 23796–23807. https://doi.org/10.1039/D1CP02802B.

(2) Stukalin, E. B.; Cai, L.-H.; Kumar, N. A.; Leibler, L.; Rubinstein, M. Self-Healing of Unentangled Polymer Networks with Reversible Bonds. *Macromolecules* **2013**, *46* (18), 7525–7541. https://doi.org/10.1021/ma401111n.

(3) Vogel, H. Temperatura Bhangigkeitgesetz Der Viskosität von Flüssigkeiten. *J. Phys. Z.* **1921**, *22*, 645–646.

(4) Fulcher, G. S. Analysis of Recent Measurements of the Viscosity of Glasses. *J. Am. Ceram. Soc.* **1925**, *8*, 339–355.

(5) Tammann, G.; Hesse, W. Die Abhängigkeit Der Viscosität von Der Temperatur Bie Unterkühlten Flüssigkeiten. *Z. Anorg. Allg. Chem.* **1926**, *156*, 245–257.

(6) Böhmer, T.; Gabriel, J. P.; Richter, T.; Pabst, F.; Blochowicz, T. Influence of Molecular Architecture on the Dynamics of H-Bonded Supramolecular Structures in Phenyl-Propanols. *J Phys Chem B* **2019**, *123* (51), 10959–10966. https://doi.org/10.1021/acs.jpcb.9b07768.
